# Charcot Neuropathic Arthropathy of the Foot: A Literature Review and Single-Center Experience

**DOI:** 10.1155/2016/3207043

**Published:** 2016-08-30

**Authors:** Tomas Kucera, Haroun Hassan Shaikh, Pavel Sponer

**Affiliations:** ^1^Department of Orthopaedic Surgery, University Hospital Hradec Kralove, Hradec Kralove, Czech Republic; ^2^Faculty of Medicine in Hradec Kralove, Charles University in Prague, Prague, Czech Republic

## Abstract

Charcot neuropathic osteoarthropathy of the foot is a relatively common complication of diabetic neuropathy. Incorrect diagnosis and improper treatment often result in the extremity having to be amputated. This paper summarises the current view on the etiology, diagnostics, and treatment of diabetic Charcot neuropathic osteoarthropathy, with particular focus on preserving the extremity through surgical intervention from our own experiences.

## 1. Introduction

Charcot neuropathic osteoarthropathy (CN) is a chronic, progressive condition of bones, joints, and soft tissues, most commonly occurring in the area of the foot and ankle as a result of peripheral neuropathy. It is characterized by a local inflammatory process in the early stages and gradual development of bone loss, joint dislocation, and fixed deformities. These deformities can secondary lead to infected ulcerations and eventually to osteomyelitis. In general, any part of skeleton can be affected.

Diabetes mellitus, together with neuropathy, is currently considered the main cause of CN. Data indicating the prevalence and incidence of the condition suggest that it often goes undiagnosed among sufferers of diabetes, with figures ranging from 0.4 to 13% among diabetics [1]. However, changes diagnosed by X-ray and corresponding with CN are detected in up to 29% of diabetics. Bilateral disability has been observed in numbers ranging from 9 to 39% of patients. When MRI is used as a diagnostic method, the detection rate rises to 75% of documented cases [2, 3]. Sohn et al. [4] state that the mortality rate is 28.3% within five years in patients with CN.

The common issue is an early diagnosis and an appropriate treatment, in case of an acute phase where it is difficult to differentiate an acute osteomyelitis. Even though the treatment of CN is mostly conservative, the surgical options might be beneficial for the patients. However, the crucial question is when, where, and how a surgical therapy has to be used.

## 2. Materials and Methods

A PubMed search was done with the key word “Charcot foot, neuropathic arthropathy, Charcot arthropathy.” We could trace about 400 up-to-date papers on the subject. Electronic database was systematically searched for literature discussing the history, pathophysiology, assessment, imaging methods, diagnosis including osteomyelitis, classification, and management of CN. We applied no restrictions on publication date. Article eligibility was assessed independently by all authors. Reasons for exclusion of articles based on title or abstract were (1) nonoriginal data (e.g., editorials, guidelines, and comments), (2) nonclinical articles (e.g., technical or animal studies), (3) case reports, and (4) articles not written in English language. All authors independently chose the most up-to-date papers with regard to target topics resulting in the identification of 59 “most pertinent” articles. Together we discussed and compared the relevant information from all these sources with our clinical practice and included them in this review.

### 2.1. History

Musgrave first described neuropathic osteoarthropathy in 1703 as an arthralgia caused by venereal disease [6]. Later, Mitchell [7] supposed the relation between spinal lesion and rheumatism of lower extremities in 1831. Charcot described the neuropathic aspect of the condition in detail in 1868 and detected spinal damage resulting from tabes dorsalis as a cause [8] and his brilliant presentation,* Demonstration of Arthropathic Affections of Locomotor Ataxy*, at the 7th International Medical Congress (1881), established this disease as a distinct pathological entity. Much later, in 1936, Jordan [9] revealed diabetes mellitus to be a possible cause of neuropathic osteoarthropathy. Nevertheless, the etiology, diagnostics, and treatment of this condition have to this day yet to be fully addressed.

### 2.2. Pathophysiology

Numerous factors contribute to the development of CN. Two main theories concerning the origin of the condition have been discussed in the past. The neurotraumatic theory is based upon damage to sensory feedback resulting from progressive destruction of bones and joints brought about by repeated trauma. The neurovascular theory highlights the changes in blood supply caused by neuropathy, most of all lesions in the sympathetic nerves which affect bone resorption [10]. The current accepted theory of CN origin states that, in susceptible individuals with peripheral neuropathy, an unregulated inflammatory process is triggered which leads to an increased expression of the polypeptide receptor activator of nuclear factor kappa-B ligand (RANKL). RANKL triggers the synthesis of the nuclear transcription factor, nuclear factor-*κβ* (NF-*κβ*), and this in turn stimulates the maturation of osteoclasts from osteoclast precursor cells. At the same time, NF-*κβ* stimulates the production of the glycopeptide osteoprotegerin (OPG) from osteoblasts. A repetitive trauma with the loss of pain sensation results in continual production of proinflammatory cytokines, RANKL, NF-*κβ*, and osteoclasts, which in turn leads to continuing local osteolysis. Another possible cause is decreased secretion of the calcitonin gene related peptide (CGRP) from damaged nervous endings. Under physiological circumstances this peptide works as an antagonist of the RANKL synthesis and at the same time is responsible for the normal integrity of the joint capsule [11]. The powerful bone anabolic Wnt/*β*-catenin pathway plays a critical role in remodeling as well as preservation of the foot skeleton in the acute and chronic stages of the disease [12]. In general, diabetes may predispose to CN occurrence through a number of mechanisms. Apart from the presence of neuropathy and possible osteopenia, these include the effects of advanced glycation end products, reactive oxygen species, and oxidized lipids, which may all enhance the expression of RANKL in diabetes [13].

### 2.3. Assessment

The diagnosis is based on patient's history, clinical examination, and imaging methods. Patients are quite often not aware of any injury as a result of their lowered perception of pain. Another triggering factor can be previous surgery of the foot [14]. Diabetic nephropathy is also associated with a higher incidence of CN [15]. Trigger factors of development of the CN can also be infections (osteomyelitis precedes CN) [16] and revascularization [17]. A necessary condition for the emergence of CN is the presence of peripheral neuropathy, in particular, diabetic distal sensitive polyneuropathy. Several methods can be used to test neuropathy. The most common is the Semmes-Weinstein 5.07/10 g monofilament. Next is the pinprick test or even the more sensitive neurometer test [18]. A small fiber-predominant neuropathy is an early manifestation of diabetic neuropathy and it can progress to distal symmetric polyneuropathy. It can be difficult to diagnose because the examination (decreased reflexes, impaired vibration, and weakness) and electrodiagnostic testing can be normal. A skin biopsy is used for this purpose. Autonomic neuropathy (a type of small fiber neuropathy) is also common and plays a key role in the development of the CN. Symptoms include gastroparesis, constipation, urinary retention, erectile dysfunction, and cardiac arrhythmias [19]. Signs of inflammation are an important sign since inflammation plays a key role in the pathophysiology of CN. Oedema, erythema, warmth, and more than a 2°C difference in local temperature in comparison to the contralateral extremity are typical symptoms of active CN and it could be difficult to differentiate them from phlegmon with osteomyelitis or from an acute attack of gout [20], especially since pain occurs in only 50% of neuropathy cases [21]. In this stage the vascular supply to the foot is still maintained though it could be difficult to palpate an arterial pulse due to prominent swelling. The eventual fracture and joint dislocation can lead to deformities, typically to rocker bottom foot with possible ulceration. During this time, critical ischaemia of the extremity is much more frequent [11]. Skin temperature measurement is the most widely used method in the assessment of the activities of the CN. With the use of a surface-sensing temperature device (infrared thermometer), temperatures are recorded in the most affected area of the foot and compared with the same areas of the contralateral foot [22].

**Figure 1:**
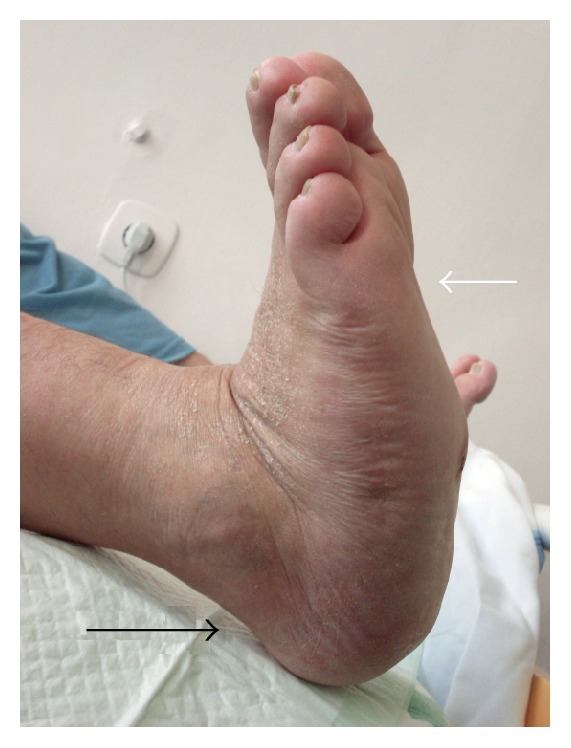
Clinical image of the right foot (CN) with contraction of m. triceps surae and plantar inclination of calcaneus (black arrow), instability of the foot, and dorsal collapse of the forefoot (white arrow).

For further prognosis and therapy it is necessary to examine the foot's stability. Instability of the forefoot can be assessed according to Assal and Stern: the pressure on the foot in the sagittal plane dorsally when the ankle joint is locked in dorsiflexion [23]. Relatively common in CN is the contraction of the triceps surae muscle which is involved in plantar inclination of the calcaneus (Figure 1).

### 2.4. Imaging Methods

Primarily it must be emphasized that the changes on the X-ray are typically delayed and have low sensitivity [24].

**Figure 2:**
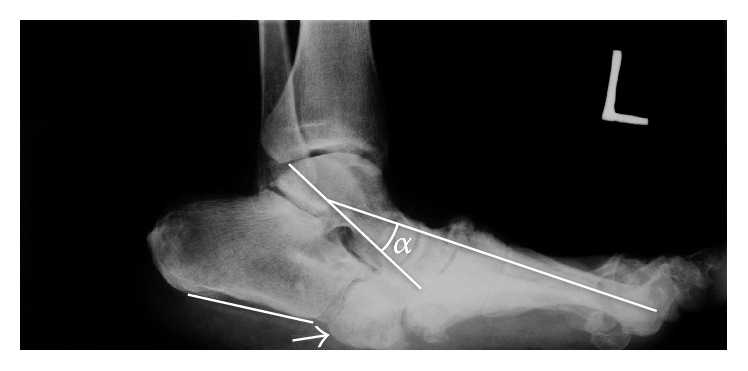
Lateral X-ray image of the weight bearing left foot, visible negative inclination of calcaneus (white line), and collapse of middle part of tarsus (angle *α*) with plantar prominence of cuboid bone (arrow).

A basic examination is an X-ray of the talus and the weight bearing foot in the anteroposterior and dorsoplantar lateral projection. During the initial stage the X-ray finding can be negative or only minor bone infractions and joint incongruence are present. In a developed stage fractures and subluxations or luxations are clearly observed. The X-ray finding depends on the specific type of CN. In a typical rocker bottom deformity a plantar dislocation of the navicular and cuboid bone is visible. A lateral projection defines inclination of the calcaneus. In CN we often find a negative inclination with a plantar tilt of the calcaneus. This deformity arises due to deformed midtarsal bones and the shortening of the Achilles tendon, which loses its elasticity during glycosylation [25]. Another finding on the lateral projection due to deformity of the middle part of the tarsus is a negative angle between the axis of talus and I metatarsus (Figure 2). The dorsoplantar projection shows changes of the position in the Lisfranc as well as Chopart joint, with resulting abduction or adduction deformity of the foot.

Examination using magnetic resonance imaging is a very valuable method for the early stages of the illness when X-ray imaging alone results in practically normal findings. An important finding is oedema of the bone marrow of two or more bones, oedema of the adjacent soft tissues, and fluid in several joints or cortical fractures. If conservative treatment is begun during this phase the condition is reversible [26].

Certain methods of nuclear medicine can be helpful not only as an alternative diagnostic method, for example, in revealing the presence of osteomyelitis, but also for monitoring the progress of the treatment. These methods, however, introduce certain difficulties. Three- or four-phase bone scintigraphy (^99m^Tc-MDP) is highly sensitive but with a low specificity. Scintigraphy with labelled leucocytes (^99m^Tc-WBC nebo ^111^In-WBC) is highly sensitive and very specific for diagnosing the infection but it is difficult to differentiate soft tissue from bone. That is why either a combination of both the abovementioned methods or FDG-PET/CT is recommended [27].

### 2.5. Diagnosis of Osteomyelitis in CN

The diagnostics of osteomyelitis in CN is difficult primarily in the active stage of the disease when clinical symptoms are practically the same both in osteomyelitis and in CN. On the contrary, in the chronic stage symptoms in osteomyelitis due to ischaemia and immunodeficiency may be masked. Diagnosing osteomyelitis is not possible on the basis of one examination alone. In general, for the differential diagnosis of osteomyelitis and CN, an important role plays a complex view of inflammatory markers and clinical manifestations of infection. The origin of osteomyelitis is in most cases caused by spread of infection from the soft tissues. History of ulceration or presence of ulceration and/or previous amputation in the area of the foot are possible factors to weigh when suspecting osteomyelitis. Osteomyelitis in CN without ulceration has a very small probability. On the contrary, a high predictive value for the presence of osteomyelitis is ulcerations bigger than two cm^2^ and deeper than three mm [28]. Another supplementary examination is probe-to-bone test (PTB). The basis of the test is whether a fine blunt steel probe can penetrate the ulceration to the bone. Sensitivity of the test is from 38 to 94% and specificity 85 to 98% [28]. Laboratory readings in CN do not show higher markers of inflammation (practically normal numbers of leucocytes, CRP, procalcitonin, and FW). Due to the chronicity of infection in osteomyelitis we can find particularly higher FW (most commonly >70 mm/hour). A basic imaging method is X-ray. Osteomyelitis shows larger osteolytic lesions and periosteal reaction when compared to plain CN. These changes are visible within two to three weeks of the onset of the infection. The abovementioned methods of nuclear medicine (four-phase bone scintigraphy, marked leucocytes, ^18^F FDG-PET, or ^67^Ga SPECT/CT) can also be used to diagnose osteomyelitis, and the range of osteomyelitic changes is also highly visible on the MRI [28]. Also ^99m^Tc-WBC SPECT/CT hybrid image is a useful tool in addition to MRI [29]. The most reliable examination is a bone biopsy, either preoperative percutaneous or peroperative, made with a biopsy needle after disinfection of the ulceration. A tissue sample is sent for microbiological and histological examination. In our clinical practice we do not use swabs to diagnose pathogens but rather send tissue samples directly. Swabs are often contaminated with normal skin flora or colonizers, and their use may result in failure to identify deep tissue pathogens [30]. For identification of pathogens, the International Working Group on Diabetic Foot (IWGDF) has proposed cultures from tissue specimens rather than from swabs [31]. However, swab cultures are less invasive than tissue biopsy or curetted tissue and swab culturing (including a vacuum transport container) may be reliable for identification of pathogens in superficial diabetic foot wounds [32].

### 2.6. Examination of Vascular Supply

Typical ischaemic symptoms like claudication and pains at rest, which would normally appear in the history, might be unrecognised because of the presence of neuropathy. During a physical examination pulsation can be impalpable and trophic changes are often found. An example of a noninvasive diagnostic method that can be also used would be measuring blood pressure in the ankle using a Doppler probe (it carries a higher risk of artificially higher pressures in the case of mediocalcinosis). In our department we use measure pressure on the big toes or transcutaneous oxygen tension (this method carries a risk of artificially lower pressures in the case of oedema) [33]. If a pathologic finding appears, we perform angiography with the possibility of revascularization. If angiography is performed postoperatively, there could be a risk of activating CN after prospective revascularization. Examination of the vascular supply must be repetitive even after surgical reconstruction of CN when a higher risk of ischaemia exists (oedema, thrombus formation of vessels). Based upon our experience, we evaluate this risk as the most significant from the point of view of possible postoperative complications.

### 2.7. Classification

The most commonly used classification according to Eichenholtz was published in 1966 (Table 1) [34].

Currently, in spite of the quite widespread usage of this classification, it is necessary to consider and search for new alternative methods of imaging. Eichenholtz evaluated X-ray images in 68 patients (altogether 94 joints) of whom only 12 were diabetic [34]. The development of examination methods of nuclear medicine and magnetic resonance have shown that there are already noticeable and recordable changes of CN even when X-ray images are negative, and starting treatment at such an early stage can prevent deformities. This stage has been labelled as stage 0 and has been added to the original classification. Alternatively, stage 1 has been divided into 1a and 1b [5]. With regard to the fact that the initial change in CN is an inflammatory reaction, which corresponds with oedema of bone marrow, a classification of CN based upon MR imaging has been suggested. It recognises two stages of the disease—active and inactive—according to the presence or absence of oedema of the bone marrow and distinguishes two grades—0 and 1—according to the presence or absence of cortical fractures. These fractures represent a worse prognosis from the point of view of developing deformities (Table 2) [5].

### 2.8. Conservative Treatment

The treatment of CN is mostly conservative. This method is based on immobilisation and the complete absence of weight bearing for the affected extremity in the active stage. There are various opinions concerning the type of immobilisation and the period of nonweight bearing for the foot. The most common immobilisation used is a total contact cast (TCC) changed three days after the initial application and then every week. Alternatively, it is possible to use Charcot Restraint Orthotic Walker (CROW) prefab orthoses. The period of fixation depends on the reduction in oedema and a drop in skin temperature below 2°C compared to the contralateral extremity [35]. The recommended length of fixation varies from six weeks to three months followed by a change of orthosis. Similarly, the recommended period without any weight bearing varies—starting from weight bearing during application of TCC to the usage of a wheelchair as a preventive means against overloading the other extremity [36]. Koller et al. [37] recommend six to eight weeks of TCC and a wheelchair with subsequent change for individual orthosis fixing the affected segment and at the same time preventing tibial rotation, thus enabling only axial weight bearing (a so-called frame orthosis). In the chronic stage of the condition, a deformed foot in plantigrade position capable of weight bearing in shoes or an orthosis without increasing deformity is suitable for conservative treatment. The type of the prosthetic equipment depends on the gravity of the deformity and on the eventual presence of ulceration. It is possible to use various types of walkers, ankle-foot orthoses, orthotic shoes, or adjusted regular shoes [38]. In general we prefer conservative treatment in both stages by a multidisciplinary team. Only after the failure of conservative management does a patient become a candidate for the surgical treatment. This treatment is beneficial in CN refractory to off-loading and immobilisation or in the case of recalcitrant ulcers [11]. A separate question is the problem of weight bearing in the case of conservatively treated or operated CN. Gait dysfunction has been proven in patients with diabetes [39]. We have the same experience as Koller et al., most of the patients suffering from peripheral neuropathy are not able to reduce weight loading of the foot in a controlled manner with the help of crutches, and there is a risk of overloading the contralateral limb and a risk of fall. Therefore we recommend full weight bearing wherein we gradually prolong the time and speed of the walk [37].

To support healing some medicaments have been used, bisphosphonates, which inhibit osteoplastic bone resorption, and intranasal calcitonin, which has had fewer complications [40]. Nevertheless, beneficial effect of pharmacological treatment (improvement of markers of resorption versus an absence of clinical marks of healing and side effects of the therapy) as well as physical stimulation of the bone growth is yet to be fully demonstrated.

### 2.9. Indication for Surgical Treatment

Besides conservative treatment, the possibilities of surgical treatment have also been looked into and the benefits and risks of such treatment have been considered. Saltzman et al. [41] evaluated retrospectively conservative treatment of 127 extremities in 115 patients over a period of 20 years. The study found that the annual rate of amputations was 2.7%, 47% of patients used an orthosis for a period longer than 18 months, and the risk of ulceration appeared in 40% of patients. Ulceration is often accompanied by a high risk of amputation. At present, specific methods for the surgical treatment of CN to save the extremity or delay major amputation are still being developed. Foot reconstruction, resection of bony prominences, and major amputations are considered for the surgical treatment (Table 3).

Major amputations in CN (generally we prefer the below-the-knee amputation) are still the current solution. If carried out properly, if the healing is complete, and if the patient is equipped in the prosthetic and rehabilitation department with a suitable prosthesis and has an adequate walking regime as part of the rehabilitation, then we know from experience that these patients, though initially perhaps unwilling to undergo surgery, are more satisfied compared to those who use orthosis for a long time, who required constant dressings of ulcerations and repeated visits to hospital. Based on our personal experiences, we use transcutaneous oxygen tension more than 35 mmHg as a predictive factor for successful healing of below-knee amputation. In dialysis patients we deal with problem of a suitable prosthesis after major amputation due to changes of extremity volume between dialyses.

Bone resections are done as a separate intervention in isolated bone prominences, mostly in cases of a high bony pressure that cannot be accommodated with orthotic and prosthetic means and in stable plantigrade foot [11]. In some cases, a Strayer procedure or Achilles tendon prolongation is necessary because of frequent cases of pes equinus in the diabetic foot. Such intervention carries a risk of instable foot in the case of larger bone resection. A bone resection is also done as preparation for reconstruction of the foot in case an infected ulceration is present or if there is suspicion of osteomyelitis.

With regard to poor bone quality and the presence of neuropathy in long-term healing, the so-called superconstruction principles for reconstruction operations have been set up: (a) extending arthrodesis beyond the affected area on neighbouring joints, (b) resection of the bone for mild shortening of the foot enabling adequate repositioning of the deformity without excessive tension of soft tissues, thus helping prevent secondary ischaemisation, (c) usage of the strongest possible implant which can be tolerated, and (d) introduction of an implant that can maximally increase mechanic stability, which is the main goal [42].

### 2.10. Types of Implants

In general we can use different types of external fixators or internal fixators according to the type of deformity and preference of the surgeon. In the case of external fixators, the most suitable enabling gradual correction seems to be ankle-foot fixators of the Ilizarov type or the Taylor Spatial Frame. Their disadvantage is the relatively high purchase cost. Mostly, a three-plane fixation that combines common types of external fixators is used. An advantage of this method is the absence of internal implant that may increase the risk of infection and the possibility of earlier weight bearing on the foot. As for the internal fixations, plates are recommended if the implantation is from the plantar side, although nowadays angle stable plates are used more often. They enable good stability in an osteoporotic bone and greater variability from the point of view of plate placement. The disadvantage is the need for a wider surgical approach and problematic healing with the exposed implant [42]. In our department we use, besides external fixators, a technique of reconstruction by axial screws—Midfoot Fusion Bolt 6.5 mm (DePuy/Synthes). When the resection is done and the reposition is finished we use them to fix intramedullary both the medial and lateral columns and subsequently apply plaster cast fixation. By using such a technique we eliminate the disadvantages of using the plates as mentioned above and we did not observe any osseous healing failure reporting by some authors [43, 44].

### 2.11. Timing of the Surgery according to the Stage of the Disease

Most of the earlier operations have been carried out only in the chronic, inactive stage. In the active stage, an inflammatory reaction with oedema and osteoporosis are present, thus increasing the risk of complicated healing. On the other hand, this stage enables easier corrections than in the fixed deformity as it is possible to use the remodeling capacity of the bone. Indications for surgery in the active stage are heavy instability, progression of the deformity, prevention of the dislocation of fragments by muscle contraction, and the general failure of conservative treatment. An external fixator is used exclusively. Usually within three to six weeks the position of the foot is gradually corrected by this external fixator into the correct plantigrade position, then an arthrodesis of joints is carried out, and the fixator is left in place for at least three more months [45]. Nevertheless, to date no sufficient relevant studies have been published demonstrating the success rate of the surgeries carried out during this active stage. In our department we carry out surgeries only in the chronic stage.

## 3. Surgical Treatment in Individual Localizations according to Sanders and Frykberg

Sanders and Frykberg classified individual localizations of CN on the foot (Table 4) [46].

### 3.1. Sanders I

**Figure 3:**
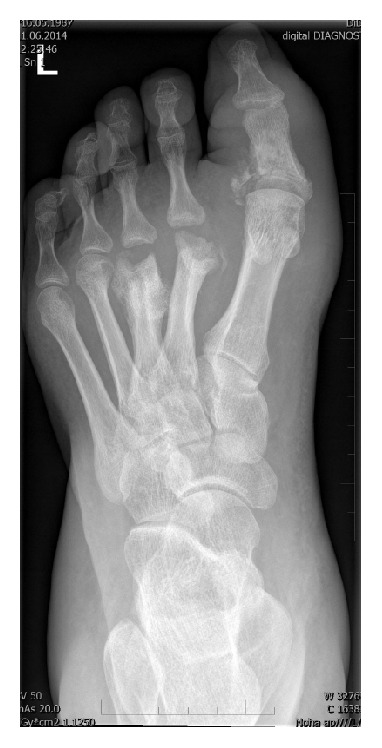
Dorsoplantar X-ray image of the left foot. Resorptive changes I–III MTP of the joints.

In this case prevailing resorptive changes were creating deformities of the metatarsal bones of the so-called candy bar type (Figure 3) [37]. This type of CN is relatively often diagnosed as osteomyelitis. Usually it is treated conservatively. In the case of dislocation in the I MTP joint we prefer arthrodesis in a revised position. Popelka recommends a fixation with the use of two screws as sufficient for this surgery [47]. On the basis of our experience we recommend using plates in CN in neuropathic terrain. In the case of heavy deformities or superimposed infection we choose bone resection. It is necessary to distinguish CN and osteomyelitis as mistaking CN for osteomyelitis may often lead to transmetatarsal amputation.

### 3.2. Sanders II

Translation of metatarsi medially or laterally is usually associated with lowering the medial column and valgus heel. A frequent consequence is abduction of the forefoot, which includes a perinavicular affection with the talus and navicular bone in plantar flection while the cuneiform bone is dislocated dorsally with the I metatarsus. The contraction of the tibialis anterior muscle worsens the deformity and practically excludes successful conservative treatment. This type of CN is very often combined with the following type III, which is why surgical treatment of both these types will be described together. Moreover, a normal position of the hindfoot is a prerequisite for the correction of the forefoot.

### 3.3. Sanders III

**Figure 4:**
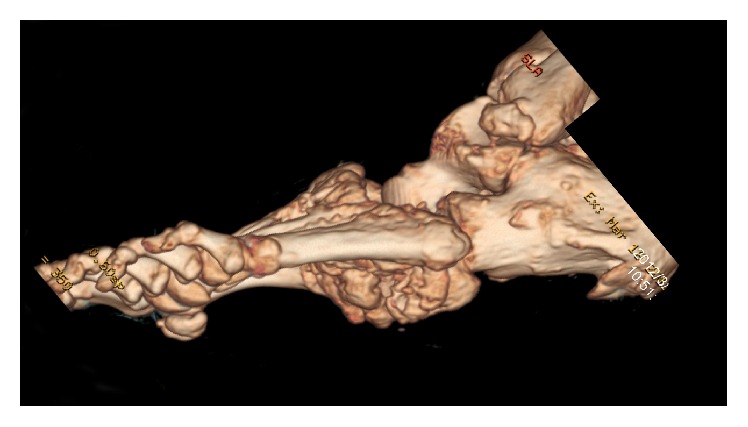
3D CT of the left foot: plantar prominence of tarsal bones and in this case plantar prominence of cuboid bone.

This is a typical rocker bottom foot with the cuboid bone in plantar prominence. This plantar bone prominence causes chronic ulcerations which do not respond to conservative therapy (Figure 4).

It is necessary to do reconstruction and stabilisation of the medial and lateral column and, in case of persistent instability, subtalar arthrodesis as well.

### 3.4. Reconstruction of the Foot

Reconstruction of the foot consists of several phases. One advantage is regional anaesthesia and minimal usage of a tourniquet. In the case of pes equinus, the first phase, according to the preoperative assessment, involves either the Strayer procedure or, more frequently, prolongation of the Achilles tendon, which can be carried out using either a Z-plasty or a technique of three mini-incisions three cm apart from each other and up to one-half of the tendon diameter (most frequently a lateral-medial-lateral incision). The position of the sole is corrected up to 90 degrees in respect to the long axis of the fibula, with the knee in full extension. This procedure restores the positive inclination of the calcaneus and facilitates the reconstruction of the medial part of the tarsus. Care must be taken to avoid pes calcaneus by overextended prolongation; this position causes heel ulcerations that do not heal, leading to the necessity for below-knee amputation. We temporarily fix the corrected position of the hindfoot with the help of the Kirschner wire from the calcaneus to the tibia.

In the next phase we make a slightly S-shaped incision on the medial side of the foot from the talus to the base of the I metatarsus, where we identify individual joint dislocations. Earlier we used the approach to the lateral column according to Ollier which led to a higher percent of secondary healing. That is why, in a deformity where the cuboid bone is the lowest bone, the callus is excised from a plantar approach. Reconstruction of the Lisfranc joint follows, and we correct abduction or adduction deformity performing osteotomy using the previously introduced Kirschner wires. We temporarily fix the corrected position with the help of Kirschner wire.

In the third phase we reconstruct the middle part of the tarsus. The navicular and cuboid bones are usually plantarly dislocated, with the goal being resectional talonavicular, naviculocuneiform, and calcaneocuboid arthrodesis in the corrected position, which is again temporarily fixed by the Kirschner wires.

**Figure 5:**
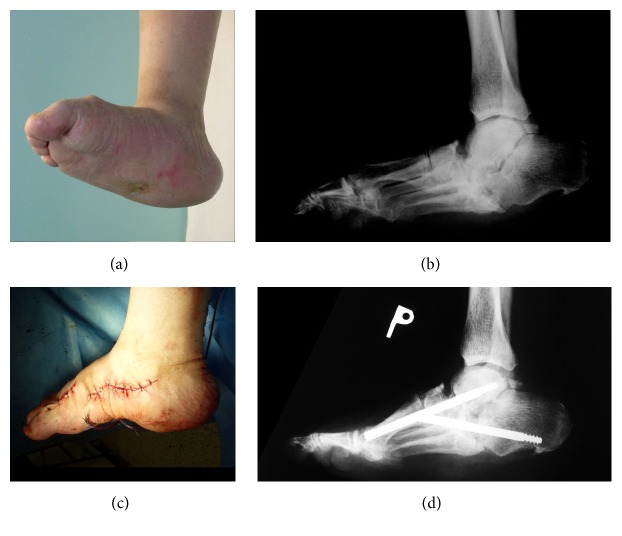
(a) Clinical image of CN with collapsed middle part of tarsus and plantar sinus (right foot). (b) Preoperative X-ray image of collapsed arch in the middle part of tarsus. (c) Clinical image immediately after reconstruction with reconstructed longitudinal arch. (d) Lateral X-ray image demonstrates reconstruction of both columns two months after surgery.

A final fixation with a Midfoot Fusion Bolt of 6.5 mm (DePuy/Synthes) is the last step. We introduce the implant medially over the head of the I metatarsus according to the deformity of the hallux from a dorsal or plantar approach up to the talus bone. Laterally we introduce the implant from the mini-incision from the area of base of the IV metatarsus through the cuboid bone to the calcaneus. To date we did not have to address residual instability between the talus and calcaneus, for which subtalar arthrodesis with the same implant is recommended [45]. We use resected bones as local autografts, reinsert tendon attachments of the medial column, and insert Redon drains and the wounds are sutured. Finally, we apply a padded plaster cast according to the type of intervention (Figure 5).

### 3.5. Sanders IV

**Figure 6:**
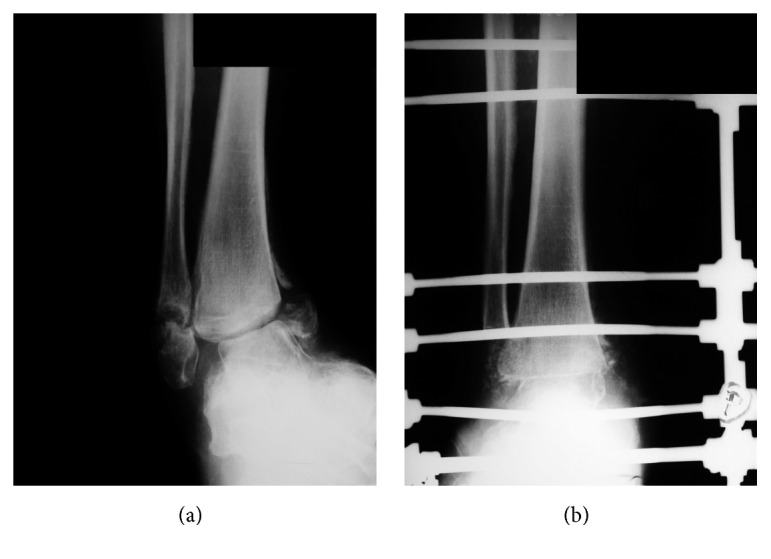
(a) AP X-ray image of the right ankle: inveterate neuropathic fractures of both malleoli. (b) A primary arthrodesis using an external fixation carried out.

In this type the ankle and frequently a subtalar joint are most affected. Because of the instability the deformity progresses and calluses and ulcerations emerge. We indicate arthrodesis of the ankle and the subtalar joint along with an external fixation (Figure 6). Healing complications have been observed by us in these cases.

**Figure 7:**
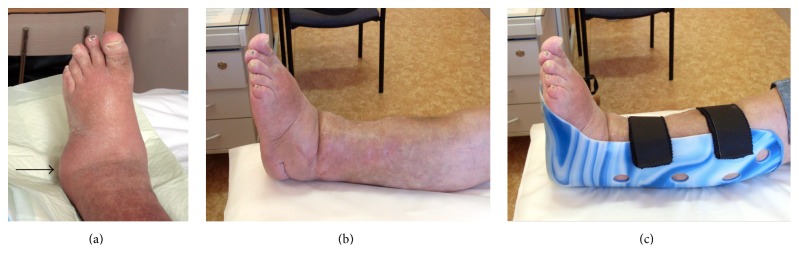
(a) A clinical image of a left foot deformity with complete dislocation of talus plantolaterally (arrow). (b) A clinical image after astragalectomy and completed healing of tibiocalcaneal fusion. (c) Individual plastic orthosis into shoes.

In the case of severe deformities we prefer astragalectomy before performing prospective transtibial amputation. After the tibiocalcaneal arthrodesis is healed it is necessary to use an orthosis for several months (Figure 7).

### 3.6. Sanders V

**Figure 8:**
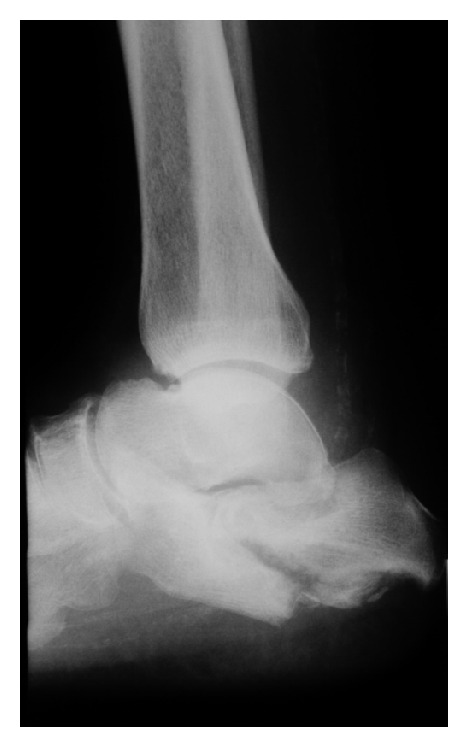
A lateral X-ray image of calcaneus: the pull of the Achilles tendon causes the fragment to be dislocated, with incongruence in the subtalar joint.

This is the least frequent type affecting the calcaneus (Figure 8). With regard to the poor quality of bone and thus to the retention of osteosynthetic material and the risk of infection, we opt for conservative treatment (orthosis). Surgical treatment is considered in the case of progressing deformity when a dislocation of fragments occurs as a result of contracting Achilles tendon. The primary goal is the stability of the hindfoot and preventing the formation of ulcerations. Part of the operative procedure involves subtalar arthrodesis.

## 4. Postoperative Care

Postoperative care depends on the type of corrected deformity, the implant used (internal or external fixation), the course of healing, whether or not the contralateral extremity is affected, and the ability of nonweight bearing on the operated extremity. In general a great deal of attention must be paid to the appropriate off-loading in the early and subacute postoperative stage and in the case of chronic CN in terms of localization. The advantage of external fixation is the possibility of earlier weight bearing; internal fixation is supplemented by a plaster cast usually for three to four months. After the external fixator or plaster cast fixation is removed, individual orthoses are applied, and weight bearing is gradually increased by reduction in limitation of walking time and speed. A suitable time for using the orthosis is up to one year. After this period individual orthopaedic shoes are usually made. A lifelong follow-up including diabetes, nutrition, and infection control by antibiotic treatment if necessary is essential.

## 5. Conclusion

The treatment of CN is mostly conservative. Thanks to new findings from the aetiopathogenesis of the condition and its biomechanics it is possible, in indicated cases, to supplement CN treatment with reconstructive procedures along with suitable implants, thus avoiding major amputation. Nevertheless, to evaluate the benefits and risks of these procedures further evidence-based studies will be necessary.

## Figures and Tables

**Table 1:** Eichenholtz classification.

Stage	Radiographic finding	Clinical finding
I development	Osteopenia, osseous fragmentation, joint subluxation or dislocation	Swelling, erythema, warmth, ligamentous laxity
II coalescence	Absorption of debris, sclerosis, fusion of larger fragments	Decreased warmth, decreased swelling, decreased erythema
III reconstruction	Consolidation of deformity, fibrous ankylosis, rounding and smoothing of bone fragments	Absence of warmth, absence of swelling, absence of erythema, fixed deformity

**Table 2:** Classification of CN based upon MR imaging. Source: [5].

Stage	Severity grade	
	Low severity: grade 0 (without cortical fracture)	High severity: grade 1 (with cortical fracture)
Active arthropathy (acute stage)	Mild inflammation/soft tissue oedema	Severe inflammation/soft tissue oedema
	No skeletal deformity	Severe skeletal deformity
	X-ray: normal	X-ray: abnormal
	MRI: abnormal (bone marrow oedema, microfractures, bone bruise)	MRI: abnormal (bone marrow oedema, macrofractures, bone bruise)

Inactive arthropathy (becalmed stage)	No inflammation	No inflammation
	No skeletal deformity	Severe skeletal deformity
	X-ray: normal	X-ray: abnormal (past macrofractures)
	MRI: no significant bone marrow oedema	MRI: no significant bone marrow oedema

**Table 3:** Indications of the surgical treatment.

Surgical treatment	Indications
Reconstruction	Stable, nonplantigrade foot Unstable foot
Resection of bony prominences	Isolated bony prominences in a stable plantigrade foot
Major amputations	Severe peripheral vascular disease Severe bone destruction including osteomyelitis Failed previous surgery

**Table 4:** Sanders and Frykberg classification.

Type	Localization
I	Metatarsophalangeal and interphalangeal joints
II	Tarsometatarsal articulations (Lisfranc)
III	Midtarsal joint line (Chopart)
IV	Ankle joint and subtalar joint
V	Calcaneus
